# Evaluation of Secondary Prevention Knowledge in Patients with Coronary Artery Disease

**DOI:** 10.3390/medicina61040693

**Published:** 2025-04-10

**Authors:** Gabriel Bálint, Zuzana Slezáková

**Affiliations:** 1 Faculty of Nursing and Professional Health Studies, Slovak Medical University in Bratislava, 833 03 Bratislava, Slovakia; zuzana.slezakova@szu.sk; 2 Department of Acute Cardiology-Coronary Care, National Institute of Cardiovascular Diseases, 833 48 Bratislava, Slovakia

**Keywords:** valuation of knowledge, secondary prevention, assessment tool, coronary artery disease

## Abstract

*Background and Objectives*: Most patients with cardiovascular disease have limited health literacy and knowledge. The promotion of knowledge among patients with coronary artery disease is an integral part of health maintenance and the minimisation of secondary cardiac events. The aim of this study was to map the percent proportion of answers and scores obtained from them in the studied domains. *Materials and Methods*: In this cross-sectional study, a Coronary Artery Disease Education Questionnaire (CADE-Q II) was used to verify knowledge gaps in the five studied domains. In total, 253 patients with coronary artery disease completed the CADE-Q II, which targeted five domains: health status, risk factors, exercise, nutrition and psychosocial risk. Data were collected between June 2021 and November 2024. *Results*: An analysis of the data found a total mean CADE-Q II score of 61.05 ± 11.42 SD out of 93 points. Our research showed that the total mean score of a group of patients corresponded to an acceptable level of knowledge. Patients in the cohort provided a total of 7843 responses for the five study domains. In total, 46.8% of patients scored all answers correctly in terms of risk factors, 49.0% in terms of nutrition, 53.1% in terms of health status, 64.4% in terms of psychosocial risk, and 65.0% in terms of exercise. *Conclusions*: The use of the CADE-Q II questionnaire, with its focus on the studied domains, verifies patient knowledge and provides a foundation for education, the provision of effective information and the promotion of secondary prevention knowledge.

## 1. Introduction

Most cardiovascular patients seeking to prevent disease and related complications require modifications to their behavioural risk factors and risk, which require a professional approach [1]. Less than 60% of patients with cardiovascular disease have limited health literacy, which is associated with a low commitment to lifestyle changes, more visits to the hospital, and a higher risk of mortality [2]. Adequate knowledge about cardiovascular disease improves a patient’s awareness of potential and existing risk factors and promotes a healthy lifestyle, which leads to better cardiovascular health [3]. Nurses play a critical role in reducing health deficits and uncertainties, raising awareness and promoting health literacy, thus aiding in prevention. They can emphasise it more through comprehensive cardiovascular risk assessment and risk factor management, providing patients with focused education and counselling, thereby changing behaviour [4,5]. Tools for assessing knowledge and identifying patients with knowledge gaps and low health literacy should be incorporated into standard health care for coronary artery disease. The CADE-Q II questionnaire is a suitable tool for evaluating both knowledge and the effectiveness of education in secondary prevention [6,7].

## 2. Study Objective

We aimed to map the percent proportion of answers and the scores obtained from them in the studied domains.

## 3. Materials and Methods

In this cross-sectional study, 253 patients hospitalised at the National Institute of Cardiovascular Diseases and Cardiac Centre in Nitra, Slovak Republic, were approached, with data collected between June 2021 and November 2024.

### 3.1. Inclusion Criteria

The age of patients hospitalised with coronary artery disease was between 35 and 75 years.

### 3.2. Exclusion Criteria

The exclusion criteria included patients with congestive heart failure, chronic liver failure, G3–G5 stage chronic kidney disease and post-cardiac arrest amnesia, as well as those who wished to opt out from this study.

### 3.3. Data Collection

The Slovak-language version of the standardised Coronary Artery Disease Education Questionnaire II was used for this study, with the participating patients therein approached separately by the study investigator. The questionnaire focused on the following five domains: health status, risk factors, exercise, nutrition and psychosocial risk. It contains 31 closed items with a choice of one out of four answers. Three points are given for a completely correct answer, one point for a partially correct answer, and none for two incorrect answers. The maximum achievable score is 93 [8].

### 3.4. Data Analysis

The questionnaire return rate was 100%, and all the items in the questionnaire were answered. Using a descriptive analysis of the obtained CADE-Q II data, the monitored variables were subsequently analysed with the statistical programme IBM SPSS 23, with continuous variables tested for normality of distribution using the Kolmogorov–Smirnov and Shapiro–Wilk goodness-of-fit tests with normal distribution. The parametric Student T test and non-parametric Mann–Whitney U test were also used. All tests were carried out at the level of significance of α = 0.05. A 95% confidence interval was used to objectively measure the dispersion of the obtained data.

## 4. Results

### Sample Characteristics

Among the patients sampled, 75.5% were male and 24.5% were female, with a mean age of 57.47 ± 9.08 SD for males and 59.64 ± 8.26 SD for females. The youngest patient was 35 years old, and the oldest was 75 years old. Other sample characteristics are shown in Table 1.

Patients in the cohort provided a total of 7843 responses for the five study domains (100%), of which 4366 responses (55.66%) scored three points, 2388 responses (30.45%) scored one point, and 1089 responses (13.89%) scored no points.

The rate of completely correct responses from the patients was 46.8% for risk factors, 49.0% for nutrition, 53.1% for health status, 64.4% for psychosocial risk and 65.0% for exercise.

Less than 60% of the patients scored 3 points for their answers to health status questions 1, 5, 6, and 7; to risk factor questions 1, 3, 4, and 5; to exercise questions 2, 4, and 7; to nutrition questions 2, 6, and 7; and to psychosocial risk questions 4 and 5.

The total score of the patients was 61.05 out of a possible 93 points, where 13.55 out of a possible 21 points were scored in the domain of health status, 9.19 out of 15 points in risk factors, 15.46 out of 21 points in exercise, 12.39 out of 21 points in nutrition and 10.46 out of 15 points in psychosocial risk.

Out of all patients who responded, 0.4% demonstrated great knowledge, 37.5% good knowledge, 52.2% acceptable knowledge, 9.5% poor knowledge and 0.4% insufficient knowledge.

## 5. Discussion

Low health literacy level is associated with adverse cardiovascular risk factor profiles and increased risk of cardiovascular disease incidence and mortality. The sample had a high prevalence of patients with arterial hypertension, and diabetes mellitus. It also included regular smokers, with smoking significantly increasing cardiovascular risk under non-optimised treatment and non-observance of cardioprotective lifestyle principles (Table 1). Traditional risk factors such as diabetes, hypertension and body mass index have been shown to mediate the relationship between education and cardiovascular diseases [9]. Although education has long been considered an essential part of cardiovascular rehabilitation and care for patients with coronary artery disease, promoting an understanding of secondary prevention strategies and compliance with treatment are also necessary [10].

**Figure 1:**
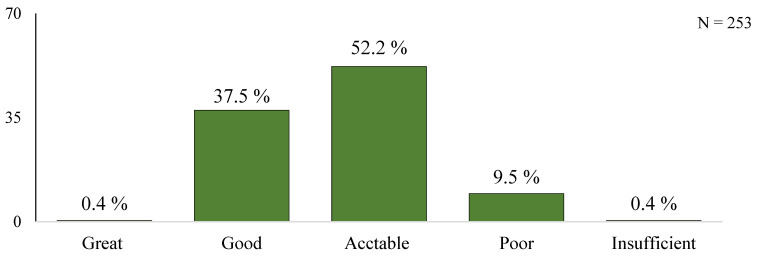
Patients’ knowledge level based on the CADE–Q II questionnaire.

Our research showed that the total mean score of a group of patients corresponded to an acceptable level of knowledge (Table 1 and Table 2) and that most of the patients demonstrated it (Figure 1). We also found that only slightly more than half of the patients gave completely correct answers in the study domains (Table 3). Promoting knowledge about health and health literacy is a factor that influences a patient’s ability to achieve and maintain a favourable health status [2]. Hospitalised patients also want and need information, including advice about their disease, its causes, its course and prognosis, treatment, necessary changes in lifestyle and necessary levels of physical activity [11].

Notwithstanding, almost half of the patients did not provide us with completely correct answers to questions about health status (Table 4), which made them feel insecure about their health and prognosis. Patients receiving either contradictory and incomplete information, or no information at all regarding their health status and treatment, experience more intense stress and a deterioration in their compliance. In addition, uncertainty or lack of knowledge in a patient with regard to their own state of health will have a negative impact on both their health and quality of life [11,12]. Our research showed that the patients scored the lowest in their answers to four of the health status questions (Table 5 and Table 6). Educating patients about treatment has been known to be associated with better knowledge, better quality of life, and lower rates of anxiety and depression [13]. Informing the patient receiving support, so that they understand the risks and benefits of each treatment alternative, is an important aspect of patient education [4].

**Figure 2:**
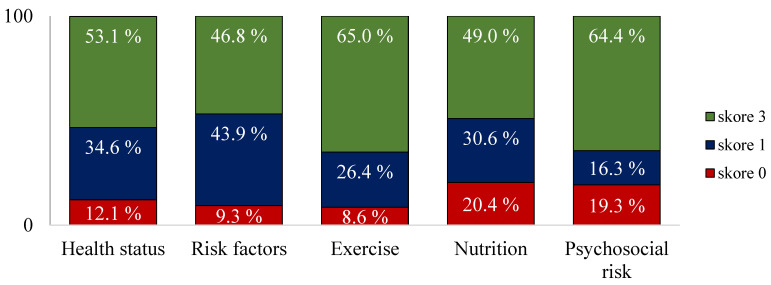
Percentage of patient responses to the CADE-Q II questionnaire; *n* = 253.

In addition, more than half of the patients did not give completely correct answers to some of the risk factor questions (Table 4). In this domain, patients gave the lowest number of completely correct answers (Figure 2), and the same results have been obtained in studies on patients with coronary artery disease [14,15]. While influencing risk factors and effective pharmacotherapy have had an impact on decreasing mortality and morbidity due to cardiovascular causes in 44% and 47% of patients, respectively [16], reductions in an individual’s risk factors should be adapted to overall cardiovascular risk, and all recognised individual risk factors should be addressed and treated during cardiovascular rehabilitation. Nurse-coordinated programmes can increase efficacy [17]. Therefore, patients should understand the specific risk factors that increase the likelihood of another cardiac event and be well aware of the health risks, including the presence of risk factors and the benefits of mitigating them [12]. Patients in this cohort scored the lowest in their answers to four of the risk factor questions (Table 5 and Table 6). The role that nurses play here is to identify those risk factors increasing the probability of a cardiac event in a patient, to intervene, and, should there be a lack of knowledge, to advocate for adherence to pharmacological and non-pharmacological treatment [18]. It is well known that active management of recognised risk factors enables any individual with coronary artery disease to achieve an optimal risk profile and better quality of life.

Our research showed that more than a quarter of patients failed to give completely correct answers to some of the exercise questions (Table 4). In this domain, patients gave the highest number of completely correct answers (Figure 2), and the same results have been obtained in studies on patients with coronary artery disease [14,15]. Even though a low level of prevention knowledge about exercise increases the risk of secondary cardiac events, regular aerobic exercise is a well- known, non-pharmacological prevention method that can mitigate it [19,20]. This should be a reason for offering therapy to any patient with coronary syndrome as part of secondary prevention, with the intensity, type and frequency of exercise being adapted to biological age, functional capacity, safety, comorbidities present, lifestyle habits and previous exercise experience [21]. Patients in the cohort scored the lowest in their answers to three exercise questions (Table 5 and Table 6). The objective behind educating patients about physical exercise is to provide them with knowledge about the benefits of physical activity, to raise awareness about the options available for increasing their levels of physical activity, and to explain the methods for overcoming barriers and negative attitudes towards it [11], since it is known that a sedentary lifestyle and lack of exercise are significant risk factors for morbidity and mortality due to cardiovascular causes [16].

Furthermore, less than half of the patients gave completely correct answers to all the nutrition questions (Table 4). Although nutrition significantly contributes to the reduction in risk factors in both primary and secondary prevention [12], the choice for coronary artery disease patients to pursue a healthy diet has been associated with improvement in the management of risk factors and the minimisation of the pathophysiological mechanisms that contribute to the incidence of secondary cardiac events. Patients in the cohort scored the lowest in their answers to three of the nutrition questions (Table 5 and Table 6). Unhealthy eating habits are significant factors contributing to progressive coronary artery disease. When providing specific dietary counselling and explaining the relevant risk factors, a cardioprotective diet would be the first step of secondary prevention [22,23]. A Mediterranean diet supplemented with olive oil has been shown to reduce the probability of acute coronary syndrome and reduce mortality in high-risk patients [24].

More than a quarter of the patients did not give completely correct answers to all of the psychosocial risk questions (Table 4). While it is known that excessive stress can constitute a real threat to health, the effects of psychosocial stress are just as dangerous as those from biological stress [25]; chronic stress activates the body’s stress response system over the long term, overexposing the body to cortisol, catecholamine and other stress hormones that can disrupt almost all of its processes [26]. Patients in the cohort scored the lowest in their answers to two of the psychosocial risk questions (Table 5 and Table 6). Bearing this in mind, we should investigate our patients’ experience and stress, concurrently educating them in stress management [19]. Patients who have experienced a cardiac event are more often exposed to psychosocial problems, and it is normal for them to show signs of depression and anxiety [12]. Along with emotional stress, these factors are associated with reduced quality of life and progressive arteriosclerotic disease [22]. Patients with depression are more prone to not comply with treatment, to skip follow-up examinations, and to lack the motivation to quit smoking, change their sedentary lifestyles, or start a healthy diet [24], thereby increasing their risk of fatal and non-fatal cardiac events.

## 6. Conclusions

CADE-Q II was used in this study to map answers and verify gaps in secondary prevention knowledge in the studied domains. The research findings revealed a low percentage of completely correct answers in the studied domains, including risk factors, nutrition, and health status, though there was a higher percentage of partly correct answers obtained from patients in the domains of risk factors and health status. Patients answered more questions completely correctly in the domains of exercise and psychosocial risk, while giving the greatest number of incorrect answers in the domains of nutrition and psychosocial risk. Our research, furthermore, revealed that patients had low scores for several questions in the study domains, and there was a low percentage of completely correct answers to many of the questions in the study domains. There needs to be more emphasis given on patient education.

The results from our research point toward the need for more effective promotion of secondary prevention knowledge in order to minimise secondary cardiac events and increase the quality of life of patients with coronary syndrome. A prerequisite for effective cardiovascular rehabilitation is a comprehensive, multidisciplinary and holistic approach. CADE-Q II validates the gaps in knowledge among the five study domains and allow practitioners to establish individually tailored, effective education plan as part of secondary prevention.

Recommendations for practice:

- Nursing with assessment tools in order to fill knowledge gaps with regard to secondary prevention among coronary artery disease patients;
- Focusing on education for coronary artery disease patients with comorbidities and behaviour that increases cardiovascular risk;
- Stressing the promotion of knowledge and secondary prevention education among patients hospitalised with coronary artery disease.

## 7. Limitations

The generalisability of the study findings is limited. A study on a larger sample of patients should be conducted in the future. Further research in this area is needed and recommended.

## Figures and Tables

**Table 1:** Sample characteristics.

**Sex**	*n*	%	**CADE-Q II** **(mean ± SD)**
**Male**	191	75.5	61.45 ± 10.94
**Female**	62	24.5	60.27 ± 12.84
**Education level**			
**University**	76	30.0	65.67 ± 10.05
**Secondary School**	173	68.4	59.48 ± 11.28
**Primary School**	4	1.6	48.50 ± 15.05
**Living area**			
**City**	170	67.2	62.11 ± 11.20
**Countryside**	83	32.8	59.21 ± 11.69
**Age groups**			
**Patients ≤ 60 years**	140	55.3	62.67 ± 11.93
**Patients ≥ 61 years**	113	44.7	59.29 ± 10.52
**Clinical Characteristics**	*n*	%	**CADE-Q II** **(mean ± SD)**
**STEMI**	139	54.5	61.17 ± 11.30
**NSTEMI**	114	45.5	61.15 ± 11.62
**CCS**	30	11.9	62.16 ± 12.04
**Indicated PCI**	183	72.3	60.87 ± 11.87
**Indicated CABG**	45	17.8	60.71 ± 11.73
**Arterial hypertension**	174	66.8	61.01 ± 11.04
**Diabetes mellitus**	56	22.1	59.66 ± 12.10
**Tobacco use**			
**Smoking profile**	*n*	*%*	**CADE-Q II** **(mean ± SD)**
**Current smokers**	93	36.8	59.52 ± 11.81
**Former smokers**	68	26.9	61.47 ± 11.46
**Never smokers**	72	28.5	63.70 ± 11.56
**Quitting smokers**	12	4.7	60.33 ± 7.42
**Occasional smokers**	8	3.2	56.0 ± 5.6
**Anthropometric characteristics (mean + SD)**			
	**Male**	**Female**	**N = 253**
**Body mass index**	28.77 ± 4.12	28.05 ± 5.99	28.59± 4.64
**Waist circumference (cm)**	104.97 ± 10.64	94.73 ± 12.11	102.63 ± 11.79
**Body Mass Index**	*n*	%	
**BMI 18.5–24.9**	59	23.3	
**BMI 25–29.9**	103	40.7	
**BMI 30–34.9**	72	28.5	
**BMI 35–39.9**	15	5.9	
**BMI ≥ 40**	4	1.6	
**Biological parameter**			
**Lipid profile**			
**N = 253**	**Mean + SD**	**Median (M_e_)**	**95% CI**
**TC^−7^**	5.25 ± 1.37	5.3	5.08; 5.42
**LDL-C^−4^**	3.55 ± 1.23	3.57	3.39; 3.7
**HDL-C^−4^**	1.17 ± 0.32	1.13	1.12; 1.21
**TG^−6^**		1.37	1.58; 2.01

STEMI—ST-Elevation Myocardial Infarction; NSTEMI—Non-ST-Elevation Myocardial Infarction; CCS—chronic coronary syndrome; PCI—Percutaneous Coronary Intervention; CABG—coronary artery bypass graft; TC—total cholesterol; LDL—low-density cholesterol; HDL—high-density cholesterol; TG—triglycerides.

**Table 2:** Patients’ knowledge level based on the CADE-Q II questionnaire.

KNOWLEDGE LEVEL	SCORE	*n*	%
Great	93–84	1	0.4
Good	83–66	95	37.5
Acceptable	65–47	132	52.2
Poor	46–28	24	9.5
Insufficient	<27	1	0.4

**Table 3:** Number of responses by patients in study domains; *n* = 253.

DOMAIN	3 Points Scored	1 Point Scored	0 Points Scored	*n*
Health status	940	617	214	1771
Risk factors	592	555	118	1265
Exercise	1151	468	152	1771
Nutrition	868	542	361	1771
Psychosocial risk	815	206	244	1265
N total	4366	2388	1089	7843

**Table 4:** Percentage of patient responses to the CADE-Q II questionnaire; *n* = 253.

DOMAIN	3 Points Scored	1 Points Scored	0 Points Scored
Health status	53.1%	34.6%	12.1%
Risk factors	46.8%	43.9%	9.3%
Exercise	65.0%	26.4%	8.6%
Nutrition	49.0%	30.6%	20.4%
Psychosocial risk	64.4%	16.3%	19.3%

**Table 5:** Percentage of patients scoring 3 points for their answers to CADE-Q II questions.

DOMAIN	Q1	Q2	Q3	Q4	Q5	Q6	Q7
Health status	45.5%	85.4%	79.8%	62.1%	38.7%	28.1%	32.0%
Risk factors	58.9%	68.0%	54.2%	34.4%	18.6%		
Exercise	80.2%	55.7%	81.0%	44.7%	68.0%	65.6%	59.7%
Nutrition	71.1%	18.6%	78.7%	71.1%	85.0%	1.6%	17.0%
Psychosocial risk	66.0%	69.2%	80.6%	54.5%	51.8%		

**Table 6:** Mean score of CADE-Q II items in studied domains.

DOMAIN	Q1	Q2	Q3	Q4	Q5	Q6	Q7	SCORE
Health status	1.72	2.64	2.53	2.22	1.76	1.43	1.25	13.55/21
Risk factors	2.17	2.29	2.03	1.4	1.3			9.19/15
Exercise	2.53	2.0	2.58	1.76	2.24	2.3	2.05	15.46/21
Nutrition	2.37	0.68	2.51	2.36	2.65	0.97	0.85	12.39/21
Psychosocial risk	2.28	2.19	2.45	1.8	1.74			10.46/15
N = 253								61.05/93

## Data Availability

The data presented in this study are available on request from the corresponding author.
